# Chorca

**Published:** 1907-03

**Authors:** W. T. Marrs


### Chorea.

This disease is an interesting study. The symptoms are too well known to here enumerate. The diagnosis is unmistakable. The only thing with which it might be confounded is disseminated sclerosis. In the latter the movements are fine and rhythmical and the disease occurs in adults. In chorea the tremors are fine and jerky, often grotesque, and the disease usually occurs in those from 6 to 15 years of age. Chorea seldom occurs after 20 years of age, except there be a marked hereditary taint of neurotic disorders. It is much more frequent in girls than in boys.

As to duration and prognosis the disease usually runs from six to twelve weeks, and in children eventuates in recovery in uncomplicated cases. One-third of the number of pregnant women who develop chorea die.

The causes are many and varied. As rheumatism is so often found co-existing with chorea many observers regard the former disease as a strong causative factor. Others contend that the majority of cases are dependent upon ocular defects, hypermetrophia and astigmatism being considered the most common causes of reflex trouble. It is a clinical fact that there is usually a hereditary predisposition. The parents are, in most cases, of neurotic type. Overwork, excessive mental strain and anything that lowers the child's vitality are also predisposing factors. Anemia and delayed menses are among the causes frequently ascribed. The irritation from worms has been known to produce this disease. The immediate or exciting causes are given prominent mention in medical literature. However, I do not believe they are of much importance. The disease has to have a beginning some time, and parents are likely to attribute the onset to fright, etc. Unless there is a condition ready to develop into the choreic state fright or shock would have very little influence in bringing it about, as nearly all children are at times subjected to things calculated to jar their nerves.

Many able men have tried vainly to discover a pathology for chorea. Some have thought it due to lesions in the brain, others have located it in certain nerve centers, while still others have thought it due to inflammatory products in the endometrium. The consensus of opinion is that chorea is a neurosis, producing marked functional disturbances for which an anatomical basis has not been discovered. At the present time it seems impossible to more than assume the existence of a nutritive disorder of the brain. We can only think of it being a functional disorder dependent upon a faulty metabolism.

In the treatment of chorea a number of remedies have been used with varying success. Among these we may mention iron, arsenic, sulphate of zinc, strychnine, gelsemium and caulophyllum. The writer has had the best results from arsenauro. It should be given in increased dosage until the point of tolerance is reached. The dose should then be gradually diminished. The patient should be given the medicinal effect of the remedy for a considerable time. If worms are suspected an anthelmintic should be given. The most nutritious foods should be given and constipation avoided. The child should be kept quiet and under kind, yet firm, restraint. She should have an abundance of sleep. A cup of hot milk, sipped at bedtime, helps to promote sleep. If rheumatism and cardiac symptoms complicate the case these may call for special attention. My treatment of these cases, as briefly outlined, has always been satisfactory.—*W. T. Marrs, M. D., in Medical Summary.*

